# RPS8—a New Informative DNA Marker for Phylogeny of *Babesia* and *Theileria* Parasites in China

**DOI:** 10.1371/journal.pone.0079860

**Published:** 2013-11-07

**Authors:** Zhan-Cheng Tian, Guang-Yuan Liu, Hong Yin, Jian-Xun Luo, Gui-Quan Guan, Jin Luo, Jun-Ren Xie, Hui Shen, Mei-Yuan Tian, Jin-feng Zheng, Xiao-song Yuan, Fang-fang Wang

**Affiliations:** State Key Laboratory of Veterinary Etiological Biology, Key Laboratory of Veterinary Parasitology of Gansu Province, Lanzhou Veterinary Research Institute, Chinese Academy of Agricultural Sciences, Lanzhou, China; Washington State University, United States of America

## Abstract

Piroplasmosis is a serious debilitating and sometimes fatal disease. Phylogenetic relationships within piroplasmida are complex and remain unclear. We compared the intron–exon structure and DNA sequences of the RPS8 gene from *Babesia* and *Theileria* spp. isolates in China. Similar to 18S rDNA, the 40S ribosomal protein S8 gene, RPS8, including both coding and non-coding regions is a useful and novel genetic marker for defining species boundaries and for inferring phylogenies because it tends to have little intra-specific variation but considerable inter-specific difference. However, more samples are needed to verify the usefulness of the RPS8 (coding and non-coding regions) gene as a marker for the phylogenetic position and detection of most *Babesia* and *Theileria* species, particularly for some closely related species.

## Introduction

The piroplasms, comprising mainly the genera *Babesia* and *Theileria*, are tick-transmitted protozoa that are highly pathogenic to ruminants, horses, pigs, dogs, cats and cattle, and in some cases, even to humans. In the vertebrate hosts, the infection usually causes fever, anemia and haemoglubinuria, and in severe cases, death [1]. Animals that recover from acute or primary infections naturally remain persistently infected, and act as reservoirs for infecting ticks. 

There are some controversial species placements such as *T. equi* and *B. microti* [2-6]. In the most thorough phylogenetic examination to date, incongruencies in the phylogenetic evolution with a taxonomically different dataset were displayed [6-9]. However, in the complex phylogenetic relationships between *Babesia* and *Theileria* spp., previous studies only relied on 18S rDNA gene [4]. The rDNA genes possess both conserved stems and variable loop regions which provide signals for different levels of phylogenetic inference [10], the 18S rDNA sequences support many piroplasm clades, but, being a slow-evolving marker it may fail to provide enough phylogenetic signal to resolve relationships at the species level such as some closely related *Babesia* species in China [11]. Internal transcribed spacer 2 (ITS2) may be a more ideal DNA barcode based on the current database for piroplasma [12], however, complex and unpredictable evolutionary behavior of ITS reduces its utility for phylogenetic analysis [11,13]. Information on mitochondrial DNA (mtDNA) from *Babesia* and *Theileria* spp., is limited, which precludes its use despite its advantage for use as a molecular marker for lower-level phylogeny [11,14,15]. Genome-wide analysis may aid in determining the taxonomy of species such as *B. microti* and *T. equi* but its usefulness as an everyday tool to classify Apicomplexan parasites is limited by availability of complete genome sequences and requirement for a large number of permutations [4-6]. It is therefore highly desirable to test other genes besides the 18S rRNA - to further improve phylogenetic analysis of *Babesia* and *Theileria* species. 

Structure and sequence signatures in ribosomal RNA and proteins are defining characteristics of the three domains of life and instrumental in constructing the modern phylogeny [16]. Based on the Ribosomal protein S8 and L4, amino acid sequence alignments of orthologous ribosomal proteins found in Bacteria, Archaea, and Eukaryota display an unusual segment or block structure with major evolutionary implications [17]. A set of 50 informative genes that could be analyzed in a broader sampling of Piroplasmida taxa to gain a greater understanding of the evolutionary relationships of the piroplasms were revealed in the comparative genomic analysis of *T. equi* [5]. Among them, we selected the 40S ribosomal protein S8 (RPS8) gene locus (including coding and non-coding regions) as phylogenetic marker to evaluate the merits and shortcomings of the phylogeny based on the comparative analysis of the RPS8 and 18S rDNA of *Babesia* and *Theileria* isolates from China for phylogenetic analysis in this study. 

## Results and Discussion

### RPS8 gene sequence information

**Figure 1:**
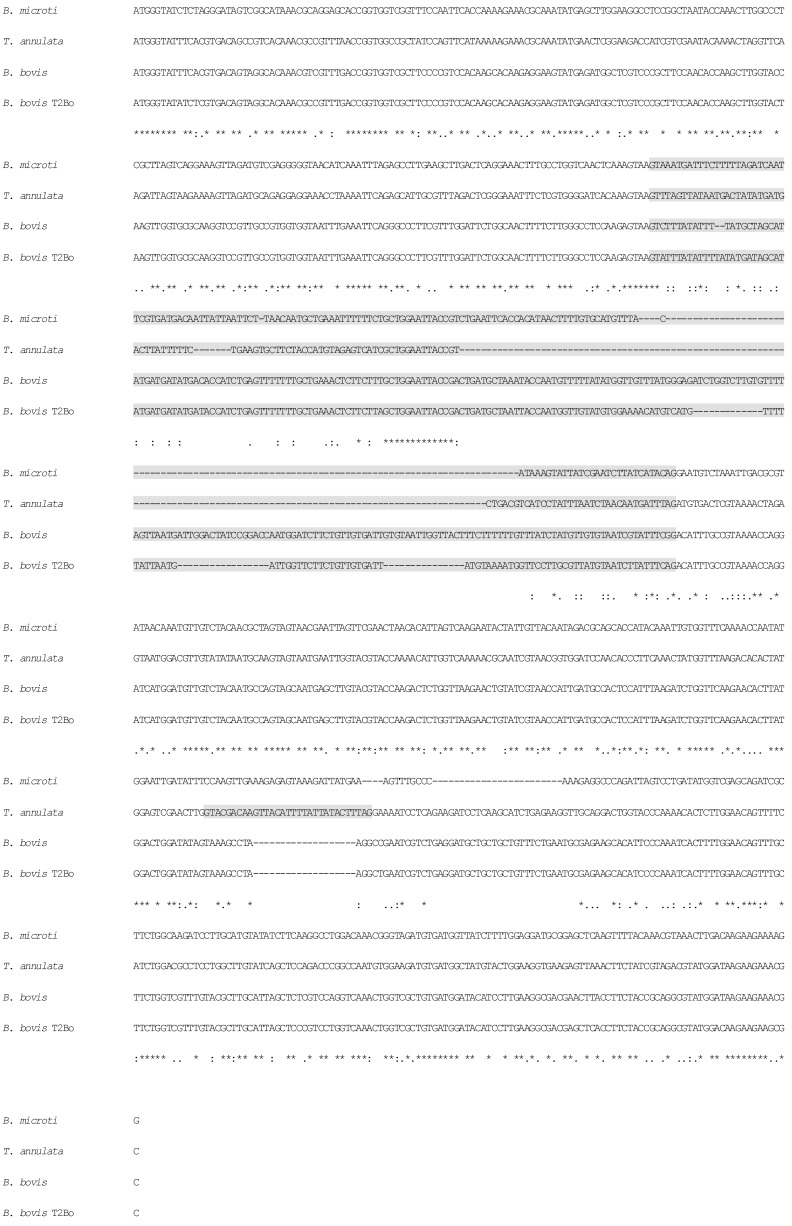
The alignment of RPS8 (coding and non-coding regions) sequences from *B. bovis* and *Theileria* species. *B. bovis* (GenBank accession no. JN400409); *B. microti* (GenBank accession no. FO082874); *T. annulata* (GenBank accession no. NC_011099); *B. bovis* (GenBank accession no. NW_001820855) were aligned using the ClustalW2 program. The non-coding region is marked with gray shading.

The complete sequences of the RPS8 (coding and non-coding regions) gene were obtained from twenty-three isolates representing seven *Babesia* and six *Theileria* spp. in China (Table 1). The primers were designed to amplify 561 bp of coding sequence for *Babesia* species and 573 bp of coding sequence for *Theileria* species, which are known to be interrupted by an intron in *Babesia* species and two introns in *Theileria* species. The size of the introns varied from 146 bp in *T. sinensis* to 282 bp in *Babesia* sp. Kashi2 and were specifically located at the conserved position in all the species (Figure 1). The identity of nucleotide sequences of RPS8 between *Babesia* and *Theileria* species varied from 63.5% to 82.9% (interspecific variability), whereas within *Babesia* and *Theileria* species (intraspecific variability) the identity was approximately 89% to 99.7% respectively (data not shown). The nucleotide sequences of the RPS8 (coding and non-coding regions) genes of *Babesia* and *Theileria* species in this study have been deposited in the GenBank database under accession Nos. JN400408 to JN4004028 and Nos. JX402859 to JX402860. The nucleotide sequences of the 18S rDNA genes of several *Theileria* species in this study have been deposited in the GenBank database under accession Nos. KF559355 to KF559357. The nucleotide sequences of the RPS8 (coding and non-coding regions) genes of *B. bovis* USA isolate (NW_001820855), *B. microti* USA isolate (FO082874), *T. annulata* Ankara strain (NC_011099), *T*.* parva* Muguga strain (NC_007345), *T. orientalis* Shintoku strain (AP011947), *T. equi* Florida strain (ACOU00000000) were drawn from the GenBank database.

**Table 1:** The host, location, vector and RPS8 and 18S rDNA gene sequences for *Babesia* and *Theileria* species used in this study, * indicated that the sequence were drawn from the database.

**parasite**	**Host**	**Location**	**Tick vector**	**Intron location**	**Genbank Accession No.**	
					**RPS8**	**18S rDNA**
*Babesia bovis*	Cattle	Shaanxian	*Rhipicephalus microplus*	212-458	JN400408	AY603398*
*B. bovis*	Cattle	Lushi	*R*. *microplus*	212-458	JN400409	JX495403*
*B. bovis*	Cattle	USA	*R*. *microplus*	212-415	NW_001820855*	-
*B*. *bigemina*	Cattle	Kunming	*R*. *microplus*	212-487	JN400410	AY603402*
*B*. *bigemina*	Cattle	Lushi	*R*. *microplus*	212-487	JN400411	JX495402*
*B. major*	Cattle	Yili	*Haemaphysalis punctata*	212-485	JN400412	AY603399*
*B. ovata*	Cattle	Lushi	*H. longicornis*	212-486	JN400413	AY603401*
*B. ovata*	Cattle	Wenchuan	*H. longicornis*	212-484	JN400414	AY603403*
*B. ovata*	Cattle	Zhangjiachuan	*H. longicornis*	212-487	JN400415	AY603400*
*Babesia* sp. Kashi2	Cattle	Kashi	*Hyalomma spp.*	212-493	JN400416	AY726557*
*B. motasi*	sheep	Lintan	*H. qinghaiensis*	212-493	JN400417	AY260181*
*B. motasi*	sheep	Ningxian	*H. longicornis*	212-491	JX402860	AY260180*
*B. motasi*	sheep	Tianzhu	*H. qinghaiensis*	212-493	JX402859	DQ159072*
*Babesia sp. Xinjiang-2005*	sheep	Kashi	*Hyalomma anatolicum*	212-403	JN400418	DQ159073*
*B. microti*	human	USA	*Ixodes scapularis*	212-362	FO082874* (2706040.2706769)	AF231348*
*Theileria annulata*	Cattle	Shanmenxia	*H*. *detritum*	212-328, 482-512	JN400419	KF559356
*T. annulata*	Cattle	Xinjiang	*H. scupense*	212-328, 482-512	JN400420	EU073963*
*T. annulata*	Cattle	Ningxia	*H*. *detritum*	212-328, 482-512	JN400428	EU083800*
*T. annulata*	Cattle	Ankara	*H*. *detritum*	212-328, 482-512	NC_011099*	-
*T. parva*	Cattle	Muguga	*Rhipicephalus appendiculatus*	212-328, 482-512	NC_007345*	HQ895968*
*T. sergenti*	Cattle	Lushi	*H. longicornis*	212-331, 485-516	JN400421	AF081137*
*T. orientalis*	Cattle	Shintoku (Japan)	*H. longicornis*	212-331, 485-516	AP011947*	-
*T. sinensis*	Yak	Lintan	*H. qinghaiensis*	212-328, 482-510	JN400422	EU274472*
*T. sinensis*	Yak	Weiyuan	*H. qinghaiensis*	212-328, 482-510	JN400423	EU277003*
*T. sinensis*	Cattle	Lintao	*H. qinghaiensis*	212-328, 482-510	JN400427	KF559355
*T*. *luwenshuni*	sheep	Ningxian	*H. qinghaiensis*	212-333, 487-515	JN400424	JF719833*
*T*. *uilenbergi*	sheep	Longde	*H. qinghaiensis*	212-329, 483-511	JN400425	JF719835*
*T. equi*	horse	Zhaoyuan	*Demacntor spp.*	212-339, 493-528	JN400426	KF559357
*T. equi*	horse	Florida (USA)	*D. variabilis ; Amblyomma cajennense ; R. microplus*	212-339, 493-528	ACOU00000000*	-

### Interspecific Genetic Distance

The ribosome, with its conserved central role in protein synthesis, has long constituted a prime subject for phylogenetic analysis [17]. The coding sequence of the RPS8 gene displays moderate conservation as a constitutive component of the ribosome. Compared with the moderate sequence identity seen in RPS8 coding regions from *Babesia* and *Theileria* species, the RPS8 non-coding regions are highly divergent. Using the Tamura–Nei model of sequence evolution, d values (Genetic Distance) were calculated independently across coding and non-coding region sequences (Data not shown). Across *Babesia* and *Theileria* species, d ranges were 0.07903 - 0.47956 (coding) and 0.12511- 1.14484 (non-coding regions). The d-values for non-coding regions are on average 2-fold greater than those for coding regions. Furthermore, the degree of variation in the RPS8 (coding and non-coding regions) dataset was considerable when observing pairwise differences between sequences. Among the eight *Babesia* and seven *Theielria* species sequenced, the average p-distances were 0.45, while for the 18S rDNA the average p-distance is 0.083, indicating the RPS8 (coding and non-coding regions) being more variable than the 18S rDNA (Data not shown). 

### Comparison of the RPS8 (coding and non-coding regions) gene-based phylogenetic trees and the 18S rDNA gene-based phylogenetic trees

**Figure 2:**
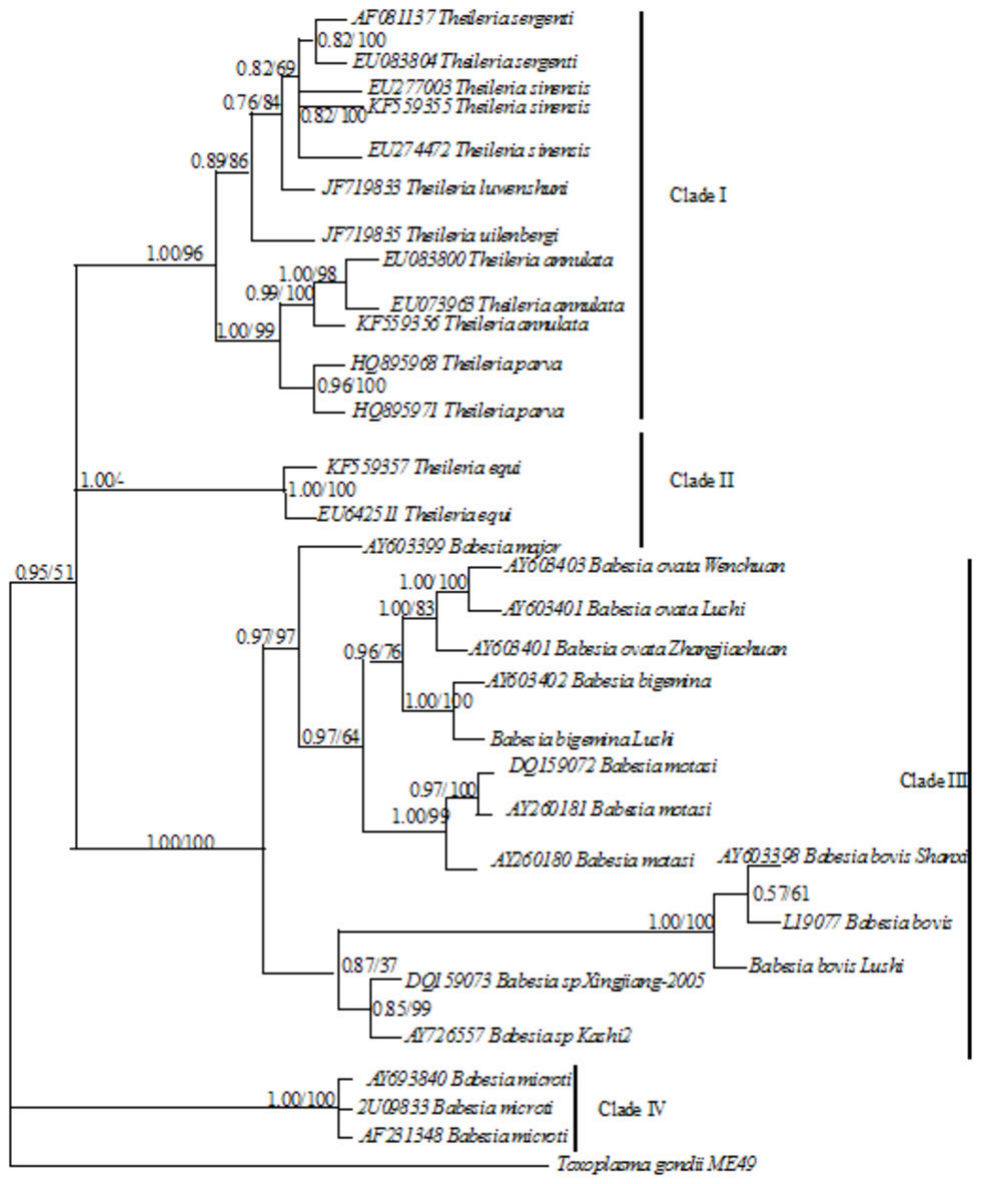
Inferred phylogenetic relationship among representative *Babesia* and *Theileria* species based on 18S rDNA sequences. The 18S rDNA sequences were analyzed utilizing Bayesian analysis (Bayes) and maximum likelihood (ML), using *Toxoplasma gondii* as outgroup. The numbers along branches indicate posterior probability (PP) and bootstrap probability (BP) values resulting from different analyses in the order: Bayes/ ML. Highly statistically supported nodes were BP≥85; PP≥0.98; while poorly statistically supported nodes were BP<60; PP<0.90.

**Figure 3:**
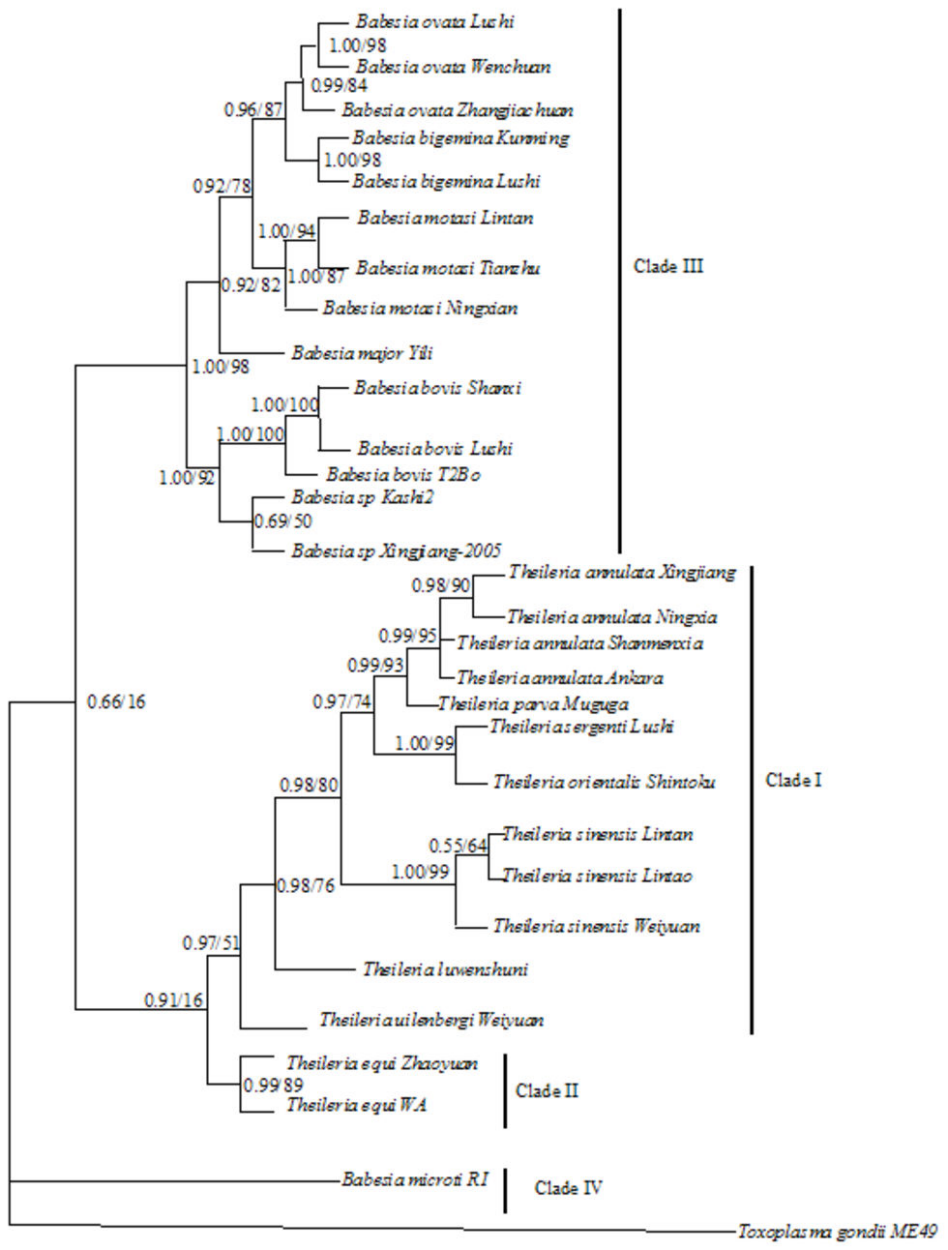
Inferred phylogenetic relationship among representative *Babesia* and *Theileria* species based on RPS8 (coding and non-coding regions) gene sequences. The RPS8 (coding and non-coding regions) gene sequences were analyzed utilizing Bayesian analysis (Bayes) and maximum likelihood (ML), using *Toxoplasma gondii* as outgroup. The numbers along branches indicate posterior probability (PP) and bootstrap probability (BP) values resulting from different analyses in the order: Bayes/ ML. The accession numbers of the isolates used in the phylogenetic tree were listed in Table 1. High statistically supported nodes had BP≥85; PP≥0.98; while poorly statistically supported nodes had BP<60; PP<0.90.

Phylogenetic trees based on the 18S rDNA and RPS8 (coding and non-coding regions) genes were constructed by the best-fit model of Bayesian and Maximum likelihood (ML) analysis (Figure 2 and 3). The Bayesian trees were congruent with those obtained under the ML criterion. Nodes receiving ≥85% bootstrap (BP) support in the Maximum likelihood analysis and/or ≥0.98 posterior probability (PP) in the Bayesian analysis were considered strongly statistically supported, and nodes receiving <60 BP and /or <0.90 PP were considered poorly supported, revealing significant levels of genetic diversity. In the RPS8 tree, the Bayesian and ML analyses returned nearly identical topologies, however, for *Theileria* species, there was less resolution in the ML analysis given that the RPS8 gene is highly variable. The datasets with significant heterogeneity result in the long-branch attraction and poor resolution at relatively deeper nodes when using the ML approach [18]. 

Both *Babesia* and *Theileria* were polyphyletic with four major clades being defined (Figure 3). *Theileria* species were clustered within clade I expect *T. equi*, this clade was strongly supported by both ML and Bayesian analyses (PP > 0.95, BP > 85). Clade II consisted of only three representatives of a single species, *T*. *equi*/*B*. *equi* (PP = 1.00, BP = 98-100), for which the phylogenetic status is controversial [2,3]. In our analysis, this clade was strongly supported as a separated taxon to other *Theileria* spp. in the 18S rDNA tree (PP = 1.00), furthermore, there are poorly supported as a sister taxon to other *Theileria* spp. with ML (BP = 16) analysis of RPS8. However, there was relatively moderate statistical support for *T. equi* as a sister taxon to other *Theileria* spp. in the Bayesian analysis (PP = 0.91). Its position relative to Clade I and Clade III comprising the majority of *Babesia* sp. was unresolved, indicating the unclear evolutionary position of *T. equi* [4,14]. In both the trees, *B. microti* was placed at the root of piroplasms, thereby, separating it from the *Babesia* and *Theileria* clades (Figure 2, 3), which is consistent with Genome-wide phylogenetic analyses suggesting a new genus for the *B. microti* group of strains [6].

Overall topology of the two trees showed similar major branching orders, beginning with the outgroup *Toxoplasma gondii* followed by polyphyletic *Theileria* and *Babesia* species. However, phylogenetic reconstructions show that, although it is the least variable, the 18S rDNA tree is more resolved than the RPS8 tree in the nodes of some clades, For example, *B. motasi* isolates were clearly separated from the branch composed of *B. ovata* and *B*. *bigemina* with statistical support (PP=0.97, BP=64) in the 18S rDNA tree as well as there was moderate statistical support (PP=0.92, BP=78) in the RPS8 tree. These data indicate that 18S rDNA and RPS8 reliably distinguishes the deeper branches among some *Babesia* species. Within *B. ovata* isolates, the phylogenetic relationship between the *B. ovata* Zhangjiachuan isolate and the Wenchuan isolate is reliable in the RPS8 tree (PP=0.99, BP=84) as showed in the 18S rDNA tree (PP=1.00, BP=83). The RPS8 gene seems to be equal to the 18S rDNA in recognizing close lineages among some *Babesia* and *Theileria* species. 

In the 18S rDNA tree, the phylogenetic relationship between *B. bovis* and other two *Babesia* species (*Babesia* sp. Xinjiang-2005 and *Babesia* sp. Kashi2) were poorly statistically supported (PP=0.87, BP=37) but the latter two *Babesia species* are closely related with high statistical support (PP=1.00, BP=95), which was not reconciled with morphological and biological data in previous studies (Figure 2) [19-24]. On the contrary, the phylogeny generated using the RPS8 gene did provide extremely strong support for the close sister-taxon relationship between *B. bovis* and the other two *Babesia* species (*Babesia* sp. Xinjiang-2005 and *Babesia* sp. Kashi2) (PP=1.00, BP=92) and unreliable sister-taxon relationship between *Babesia* sp. Xinjiang-2005 and *Babesia* sp. Kashi2 (PP=0.69, BP=50) (Figure 3). This is probably due to the use of a small dataset and two markers with different modes of evolution. Thus, for more accurate definition of certain *Babesia* isolates, it may be necessary to incorporate more markers that have differing evolutionary rates.

For *Theileria* species, clade I in the 18S rDNA tree was composed of two subclades, one subclade comprising *T. annulata* isolates and *T. parva*, with the other subclade consisting of *T*. *uilenbergi*, *T*. *luwenshuni*, *T. sinensis* isolates and *T. sergenti*. The phylogenetic status of two subclades resolved with strong statistical support (PP=1.00, BP=96). Furthermore, *T. annulata* and *T. parva* were closely related with a strong statistical support (PP=1.00, BP=100). *T*. *luwenshuni* formed a separate branch that is distantly related to *T. sergenti* and *T. sinensis* with statistical support (PP=0.76, BP=84) (Figure 3). *T*. *uilenbergi* formed a separate branch with statistical support (PP=0.89, BP=86). In the RPS8 tree, *T*. *luwenshuni* formed a separate branch with strong statistical support (PP=0.98, BP=76) [25,26]. 

In short, 18S rDNA as a slow-evolving marker more reliably distinguishes deeper branches among some *Babesia* species than the RPS8 gene. However, the RPS8 gene seems to be equal to the 18S rDNA in recognizing lineages among some closely related *Babesia* and *Theileria* species. 

### Combined analysis

**Figure 4:**
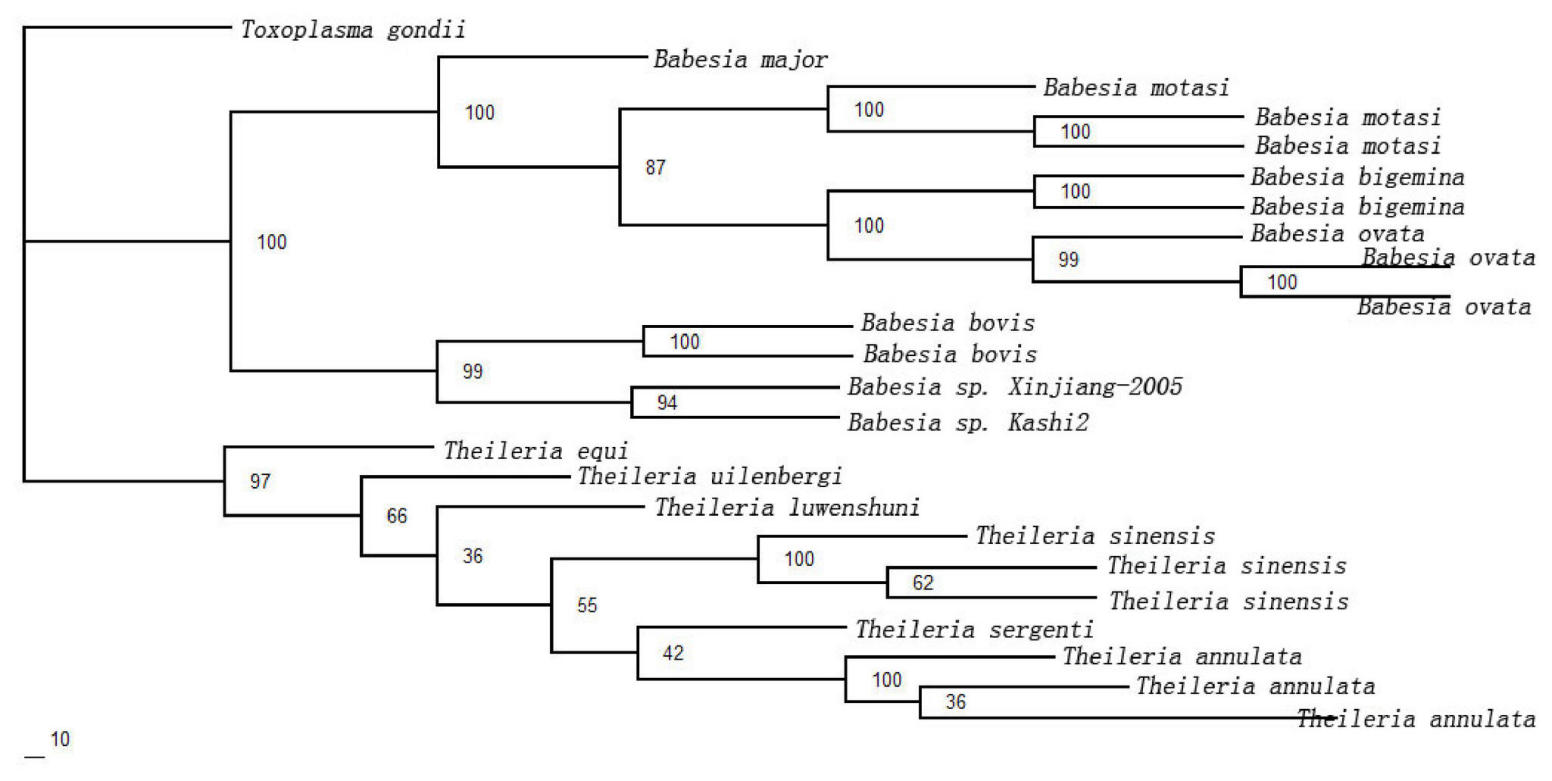
Inferred phylogenetic relationship among representative *Babesia* and *Theileria* species based on the combined data of 18S rDNA and RPS8 (coding and non-coding regions) gene sequences. The combined data of 18S rDNA and RPS8 (coding and non-coding regions) gene sequences were analyzed utilizing maximum likelihood (ML), using *Toxoplasma gondii* as outgroup. The numbers along branches indicate bootstrap probability (BP) values. The accession numbers of the isolates used in the phylogenetic tree were listed in Table 1. High statistically supported nodes had BP≥85; while poorly statistically supported nodes had BP<60.

A phylogenetic tree was reconstructed based on the combined dataset of the two markers. The resulting tree is presented in Figure 4. It is mostly reconciled with the phylogenetic trees constructed based on the RPS8 gene and 18S rDNA apart from the phylogenetic relationship of *T. equi* to other *Theileria* spp. The clade of *T. equi* was strongly supported as sister taxon to other *Theileria* spp. with ML (BP=97) analysis of the combined dataset of the two markers. However, the phylogenetic tree constructed based on the combined dataset of the two markers is not reliable with poor statistical support (Data not shown). 

## Materials and Methods

### Ethics statement

This study was approved by the Animal Ethics Committee of the Lanzhou Veterinary Research Institute, Chinese Academy of Agricultural Sciences. All sheep and calves, were handled in accordance with good animal practices required by the Animal Ethics Procedures and Guidelines of the People's Republic of China.

### Animals

All sheep and calves were purchased from a *Babesia* and *Theileria*-free area and maintained in an isolated stable. All sheep and calves were splenectomized and treated with antibiotics to promote the healing of wounds one month before the study. During this time, blood films were taken weekly from the ears of the sheep and calves to be examined by Giemsa stain for the presence of haemoprotozoan parasites. The experimental animals were tested by PCR with the universal primers for *Babesia* and *Theileria* species based on the 18S rDNA sequences prior to use to ensure that they were free of hemoparasites. After collecting blood containing parasites, the experimental animals were treated with anti-piroplasmosis drug and they were rehabilitated.

### Parasite species

The isolates used in this study are listed in Table 1. *Babesia bovis* (Shanxian and Lushi) [27], *B*. *bigemina* (Kunming and Lushi) [28], *B. major* (Yili) [29], *B. ovata* (Wenchuan and Lushi and Zhangjiachuan) [30,31,32], *Babesia* sp. Kashi2 (Kashi) [24], *B. motasi* (Ningxian and Tianzhu and Lintan) [33,34], *Babesia* sp Xingjiang-2005 (Kashi) [21], *Theileria annulata* (Xingjiang and Ningxia and Shanmenxia) [30,35], *T. sergenti* (Lushi) [36], *T. sinensis* (Weiyuan and Lintan and Lintao) [37,38], *T*. *uilenbergi* (Longde) [26], *T*. *luwenshuni* (Ningxian) [26], *T. equi* (Zhaoyuan) [39]. The reference parasite species are as follows: *T. annulata* (Ankara strain) [40], *T. orientalis* (Shintoku strain) [41], *T. equi* (USDA strain) [5], *T. parva* (Muguga strain) [42], *B. microti* (RI isolate) [6], *Toxoplasma gondii* (ME49 strain) [43], *B. bovis* (T2Bo strain) [44].

### DNA extraction

Sheep and calves were infected intravenously with 15 ml of cryopreserved infected blood stock of these *Babesia* and *Theileria* isolates. Daily post-infection rectal temperatures were measured to monitor for disease and blood smears were examined to monitor for presence of piroplasms. When the parasitemia reached more than 5% of whole blood, blood was collected into heparinised tubes. Parasite DNA was isolated using a genomic DNA Purification Kit (Gentra, USA) according to the manufacturer’s instructions. The amount of DNA isolated was assessed spectro-photometrically. Control DNA was isolated from venous blood of uninfected sheep and calves.

### PCR, cloning, and DNA sequencing

The partial RPS8 gene was amplified from the genomic DNA extracted from each sample except *Babesia microti* by conventional PCR using forward primer 5’- ATGGGTATT(A/C)TCA(G/T/)C(A)GT(C/G)GAC(T)AG-3’ and reverse primer 5’- GCGTTTCTTCTTA(G)TCCATACG -3’. The reaction mixture consisted of 10×PCR buffer, 1.5 mM MgCl_2_, 200 mM each deoxynucleotide triphosphate, 40 pmol each primer, 1.5 U of Taq polymerase (Takara) and approximately 10 ng of DNA, in a final volume of 50 ml. Each PCR consisted of 35 cycles of denaturation at 94°C for 45 s, annealing at 52°C for 60 s, and extension at 72°C for 60 s; an initial denaturation step consisting of incubation at 94°C for 5 min and a final extension step consisting of incubation at 72°C for 10 min was also included. After PCR amplification, the PCR fragment was cloned into the pGEM-T Easy vector (Promega) according to the manufacturer’s recommendations, At least two positive clones from each sample were sequenced by using ABI PRISMTM 377XL DNA sequencer (TaKaRa). All new data has been deposited in GenBank.

### Bioinformatic study of genetic distances and phylogenetic analysis

Both ML and Bayesian approaches were used to evaluate each of the individual loci separately. The outgroup taxon *Toxoplasma gondii* was used for all loci. ML analyses were conducted with PhyML [45] as implemented within Geneious [46], using the best-fit models of nucleotide substitution detected by jModelTest. Support for nodes was estimated by analyzing 1000 bootstrap pseudoreplicates for each locus. Bayesian phylogenetic analysis was conducted on the total dataset using MrBayes v3.1.2 [47]. Akaike information criterion was used to identify the most appropriate model of nucleotide substitution for the Bayesian analysis in the program MrModeltest v2.2, and the best-fit model determined by jModelTest for 18S and RPS8 was GTR with both a proportion of invariable sites (I) and variation among sites (G). The Bayesian analysis was run for 15,000,000 generations with phylogenies sampled every 1,000 generations, and values for the substitution model parameters were not defined a priori, but were treated as unknown variables with uniform priors. Resulting burn-in values (the point at which the model parameters and tree scores reached stationarity) were determined empirically by evaluating likelihood scores. The nucleotide distance matrices were created under a ML correction in MEGA 4.0. The extent of sequence disparity between specimens was calculated by averaging pairwise comparisons of sequence differences across all specimens [48].

The sequence data were also examined using Maximum likelihood analyses in PhyML [45]. The combined data of 18S rDNA and RPS8 (coding and non-coding regions) gene sequences from *Toxoplasma gondii* was used as an outlier group. The phylogenetic trees were constructed based on the combined data of 18S rDNA and RPS8 (coding and non-coding regions) gene sequences from *Theileria* and *Babesia* species determined in our laboratory or obtained from the GenBank database.

The program Genescan for predicting the locations and exon-intron structures of genes in genomic sequences from a variety of organisms (http://genes.mit.edu/GENSCAN.html). The RPS8 deduced amino acid sequence was analyzed with the Expert Protein Analysis System (http://us.expasy.org/). Theoretical molecular weights and isoelectric point (pI) were determined using Peptide-Mass (http://us.expasy.org/tools/peptide-mass.html), and prediction of potential phosphorylation sites was carried out at the NetPhos website. Identification of other motifs was conducted using the Motif scan program (http://hits.isb-sib.ch/cgi-bin/PFSCAN).
